# Global convergence in a hybrid conjugate gradient projection method for finding solutions of constrained nonlinear equations with applications

**DOI:** 10.1371/journal.pone.0335265

**Published:** 2025-10-28

**Authors:** Yan Xia, Dandan Li, Songhua Wang

**Affiliations:** 1 School of Artificial Intelligence, Guangzhou Huashang College, Guangzhou, Guangdong, China; 2 School of Mathematics, Physics and Statistics, Baise University, Baise, Guangxi, China; Khalifa University of Science and Technology, UNITED ARAB EMIRATES

## Abstract

In this paper, a hybrid conjugate gradient projection method for finding solutions of constrained nonlinear equations is proposed by integrating both hyperplane projection and hybrid techniques. The key features of this method are as follows: (1) It is characterized by a low storage requirement and relies solely on function values; (2) The designed search direction ensures the sufficient descent property without the need for line search approaches; (3) Under certain reasonable assumptions, the global convergence of the method is established; (4) Experimental results demonstrate that the proposed method outperforms the two existing methods about 75.71%, 85.36%, and 86.43% of benchmark problems in terms of CPU time, the number of function evaluations, and iterations. Furthermore, it is applied to successfully solve the sparse signal restoration problems.

## Introduction

Numerical applications for nonlinear equations span various domains, including financial forecasting [1], compressed sensing problems [2], subproblems in generalized proximal methods with Bergman distances [3], machine learning [4], and robot arm control [5,6]. Motivated by these applications, the goal of this paper is to finding solutions of constrained nonlinear equations:

e(x)=0,x∈E, (1)

where $E\subseteq {\mathbb{R}}^{n}$ is a closed convex and non-empty set, and $e:{\mathbb{R}}^{n}\to {\mathbb{R}}^{n}$ is a continuous and monotone function. Throughout the paper, we denote $\left\|e(x_{k})\right\|$ as $\left\|e_{k}\right\|$, and ‖·‖ as the Euclidean norm on ${\mathbb{R}}^{n}$. To establish a foundation for the discussion, we now review some key concepts related to nonlinear equations:

(1) A function e(·) is said to be monotone if:

$${\left(e(x)-e(y)\right)}^{\text{T}}(x-y)\geq 0,\;\forall x,y\in {\mathbb{R}}^{n}.$$ (2)

(2) A function e(·) satisfies the Lipschitz continuity condition with *L* > 0 if:

$$\|e(x)-e(y)\|\leq L\|x-y\|,\;\forall x,y\in {\mathbb{R}}^{n}.$$ (3)

Since conjugate gradient methods [7,8] are known for their simplicity and low storage requirements, they are widely used to solve the problem (1). The sequence of iterates {*x*_*k*_} generated by these methods is updated as:


$$x_{k+1}:=x_{k}+v_{k}d_{k},\;k\geq 0,$$

where $v_{k}$ is the step-size determined by a line search approach, and *d*_*k*_ is the search direction. Various methods can be employed to generate the search direction *d*_*k*_, such as Newton methods [9–11], quasi-Newton methods [12,13], Levenberg-Marquardt methods [14,15], and their modifications. For example, in [11], Waziri et al. proposed a method that approximates the Jacobian matrix as a diagonal matrix, significantly reducing storage requirements, computational cost, and CPU time. In [12], Ai-Baali et al. explored the properties of quasi-Newton methods, originally introduced by Broyden as an alternative to Newton’s method and later extended to nonlinear equations. In [14], Ou and Lin presented a hybrid method for solving non-smooth equations with box constraints, combining the Levenberg-Marquardt-like techniques and non-monotone strategies. Their method demonstrates both global and local superlinear convergence with promising numerical results. Despite the advantages of these methods, they generally require solving linear equations using either the Jacobian matrix or its approximation at each iteration, which makes them less suitable for large-scale nonlinear problems. Consequently, many researchers have turned to conjugate gradient methods for generating the search direction, as these methods do not rely on such costly operations and can efficiently handle large-scale equations.

Recently, the hybridization of conjugate gradient methods [16,17] has emerged as an interesting and effective technique for designing search directions. Several researches have explored different combinations of conjugate parameters to improve performance across various applications. For example, in [18], Waziri et al. applied the convex combination technique to develop an improved search direction based on Fletcher-Reeves (FR) and Polak-Ribière-Polyak (PRP) conjugate parameters. Similarly, in [19], Sun et al. introduced a hybrid conjugate gradient-based projection method that combines Hestenes-Stiefel (HS) and Dai-Yuan (DY) conjugate parameters. In another approach, Yusuf et al. [20] proposed a hybrid method for solving large-scale nonlinear equations, which merges FR and PRP parameters. Additionally, in [21], Danmalam et al. developed a hybrid conjugate residual method for nonlinear monotone equations with convex constraints, combining FR and a new residual parameter to ensure descent direction without line search approaches. In [22], Koorapetse et al. proposed a hybrid conjugate gradient projection method for large-scale nonlinear monotone equations, ensuring descent property and global convergence. Furthermore, in [23], Li et al. presented a hybrid conjugate gradient-based projection method with modified PRP and DY parameters, and proved global convergence and superior efficiency in numerical experiments, particularly in sparse signal and image restoration. In [24], Li et al. also developed a derivative-free descent method, which incorporates spectral gradient methods, projection techniques, and monotone line searches. In a different approach, Abubakar et al. [25] proposed a hybrid three-term conjugate gradient method that ensures sufficient descent and trust region properties without line search approaches. Lastly, in [26], Yin et al. introduced a hybrid three-term conjugate gradient projection method with adaptive line search for large-scale nonlinear monotone equations with convex constraints. These diverse hybrid methods highlight the growing potential and versatility of conjugate gradient methods in optimization, particularly for large-scale and constrained problems.

Motivated by the works in [27–29], we adopt the hybridization techniques to modify a novel conjugate parameter, which is designed to ensure the global convergence of the method. Based on this parameter, we then propose a search direction that satisfies the sufficient descent property. By incorporating effective line search approaches and projection techniques, we propose a hybrid conjugate gradient projection method specifically tailored for finding solutions of constrained nonlinear equations.

## Algorithm

To establish a coherent framework for formulating the search direction in constrained optimization, we first review the foundational works in unconstrained optimization, specifically addressing the problem of minimizing a function *f*(*x*), where $x\in {\mathbb{R}}^{n}$. The conjugate gradient method, a widely-used iterative approach in unconstrained optimization, depends heavily on the choice of conjugate parameters that govern the search direction. Hager et al. [27] developed a novel conjugate parameter, which is defined as:


$$\beta_{k}^{\text{HZ}}=\frac{g_{k}^{\text{T}}y_{k-1}}{d_{k-1}^{\text{T}}y_{k-1}}-t\frac{\|y_{k-1}{\|}^{2}g_{k}^{\text{T}}d_{k-1}}{{\left(d_{k-1}^{\text{T}}y_{k-1}\right)}^{2}},$$

where $g_{k}:=\nabla f(x_{k})$ is the gradient of the function at the current point *x*_*k*_, $y_{k-1}=g_{k}-g_{k-1}$, and $t>\frac{1}{4}$. Similarly, Yu et al. [28] developed a modified version of the PRP conjugate parameter:


$$\beta_{k}^{\text{MPRP}}=\frac{g_{k}^{\text{T}}y_{k-1}}{\|g_{k-1}{\|}^{2}}-t\frac{\|y_{k-1}{\|}^{2}g_{k}^{\text{T}}d_{k-1}}{\|g_{k-1}{\|}^{4}},$$

where the first term promotes the classical PRP conjugacy, while the second term introduces a correction to account for the history of the gradients, providing a more adaptive search direction. Moreover, Li et al. [29] presented a modified Liu-Story (LS) conjugate parameter, which is expressed as:


$$\beta_{k}^{\text{MLS}}=\frac{g_{k}^{\text{T}}y_{k-1}}{-g_{k-1}^{\text{T}}d_{k-1}}-t\frac{\|y_{k-1}{\|}^{2}g_{k}^{\text{T}}d_{k-1}}{{\left(g_{k-1}^{\text{T}}d_{k-1}\right)}^{2}}.$$

Notably, all of these conjugate parameters share a common structural pattern, which suggests that their forms can be unified into a more general framework for optimization. Motivated by these findings, we extend these conjugate parameters to address constrained optimization problems, particularly those involving nonlinear equations as in problem (1). This insight leads us to propose a hybrid conjugate parameter to ensure the global convergence of the method:

$$\beta_{k}^{\text{HCGP}}=\frac{e_{k}^{\text{T}}u_{k-1}}{o_{k-1}}-\chi \frac{\|u_{k-1}{\|}^{2}e_{k}^{\text{T}}d_{k-1}}{o_{k-1}^{2}},$$ (4)

where $e_{k}:=e(x_{k})$, $u_{k-1}=e_{k}\;-\;e_{k-1}$, $o_{k-1}=\mu \|d_{k-1}{\|}^{2}\;+\;\max\left\{d_{k-1}^{\text{T}}u_{k-1},\|e_{k-1}{\|}^{2},-e_{k-1}^{\text{T}}d_{k-1}\right\}$, and χ,μ>0. To ensure that the search direction satisfies the sufficient descent property, we define the following search direction:

$$d_{k}=\left\{ \begin{array}{cc} -e_{k}, & k=0,\\ -\left(1+\beta_{k}^{\text{HCGP}}\frac{e_{k}^{\text{T}}d_{k-1}}{\|e_{k}{\|}^{2}}\right)e_{k}+\beta_{k}^{\text{HCGP}}d_{k-1}, & k\geq 1. \end{array}\right.$$ (5)

In this section, we propose a hybrid conjugate gradient projection (HCGP) method to find solutions of constrained nonlinear equations. To ensure clarity and precision, we first discuss the line search strategy employed in our method, as well as the projection operator used to enforce the feasibility of iterates.

We adopt a line search strategy based on the approach outline in the work [30]. This approach determines the optimal step-size $v_{k}$ through the following inequality condition:

$$-e(x_{k}+v_{k}d_{k}{)}^{\text{T}}d_{k}\geq \sigma v_{k}\|d{\|}^{2},$$ (6)

where σ>0 is a small constant, and the step-size is selected according to the rule $v_{k}=\max\{\kappa t^{i}:i=0,1,2,\ldots ,\}$ with parameters κ>0 and t∈(0,1). This approach ensures a gradual and controlled descent along the search direction while maintaining stability in the optimization process.

A critical aspect of constrained optimization is ensuring that each iterative point lies within the feasible region *E*. To address this, we define the projection operator *P*_*E*_ that projects any point $x\in {\mathbb{R}}^{n}$ onto the feasible set. This operator minimizes the Euclidean distance between *x* and the feasible region *E*:


$$P_{E}[x]=\arg\min\left\{\|x-y\|\;|\;y\in E\right\},\;x\in {\mathbb{R}}^{n}.$$

The projection operator exhibits a well-known non-expansive property, which ensures that the distance between projections of two points is no greater than the distance between the original points themselves:

$$\|P_{E}[x]-P_{E}[y]\|\leq \|x-y\|,\;\forall x,y\in {\mathbb{R}}^{n}.$$ (7)

With the line search strategy and projection operator, our method computes the trial point $w_{k}=x_{k}+v_{k}d_{k}$ at each iteration. If the trial point *w*_*k*_ does not lie within the feasible region *E*, we update the next iterative point *x*_*k* + 1_ using the following update rule:

$$x_{k+1}=P_{E}\left[x_{k}-\gamma \theta_{k}e(w_{k})\right],\;\theta_{k}=\frac{e(w_{k}{)}^{\text{T}}(x_{k}-w_{k})}{||e(w_{k})|{|}^{2}},$$ (8)

where γ∈(0,2) is a scaling factor.

The iterative process continues until the term $\left\|e_{k}\right\|$ falls below a given threshold *δ*, indicating convergence to a solution. The detailed steps of the method are summarized in Algorithm 1.

**Algorithm 1. HCGP algorithm.**

1: **Initialization:** Parameters: $x_{0}\in {\mathbb{R}}^{n}$, χ,μ>0, κ,σ>0, t∈(0,1),

γ∈(0,2), δ>0. Set k:=0.

2: **while** $\|e_{k}\|>\delta$ **do**

3:   Compute the search direction *d*_*k*_ by (5).

4:   Determine the step-size $v_{k}$ by (6) and compute the trial

point $w_{k}=x_{k}+v_{k}d_{k}$.

5:   **if** $w_{k}\in E$ and $||h(w_{k})||\leq \delta$ **then**

6:    Break.

7:   **else**

8:    Update the next iteration *x*_*k* + 1_ by (8).

9:   **end if**

10:   Set k:=k+1.

11: **end while**

## Analysis of convergence properties

In order to demonstrate that the sequence {*x*_*k*_} generated by the HCGP method converges to its optimal solution of the problem (1), we must establish the following general assumptions:

(A1) The solution set $E_{*}$ of the problem (1) is non-empty;

(A2) The function *e*(*x*) is both monotone and Lipschitz continuous on ${\mathbb{R}}^{n}$.

### Search direction analysis

With these assumptions in place, we proceed to analyze the properties of the search direction sequence {*d*_*k*_}, which is a key element in proving convergence. The following lemma characterizes the sufficient descent property of the search direction sequence.

**Lemma 1.** *The search direction sequence* {*d*_*k*_} *satisfies the sufficient descent property for any* k≥0:

$$e_{k}^{\text{T}}d_{k}=-\|e_{k}{\|}^{2}.$$ (9)

*Moreover, for* k≥0*, we have:*

$$\|e_{k}\|\leq \|d_{k}\|.$$ (10)

*Proof*: When *k* = 0, we have $e_{0}^{\text{T}}d_{0}=-\|d_{0}{\|}^{2}$. When k≥1, multiplying both sides of (5) by *e*_*k*_, we have:


$$\begin{array}{ccc} e_{k}^{\text{T}}d_{k} & = & -\left(1+\beta_{k}^{\text{HCGP}}\frac{e_{k}^{\text{T}}d_{k-1}}{\|e_{k}{\|}^{2}}\right)\|e_{k}{\|}^{2}+\beta_{k}^{\text{HCGP}}e_{k}^{\text{T}}d_{k-1}\\ & = & -\|e_{k}{\|}^{2}-\beta_{k}^{\text{HCGP}}\frac{e_{k}^{\text{T}}d_{k-1}}{\|e_{k}{\|}^{2}}\|e_{k}{\|}^{2}+\beta_{k}^{\text{HCGP}}e_{k}^{\text{T}}d_{k-1}\\ & = & -\|e_{k}{\|}^{2}-\beta_{k}^{\text{HCGP}}e_{k}^{\text{T}}d_{k-1}+\beta_{k}^{\text{HCGP}}e_{k}^{\text{T}}d_{k-1}\\ & = & -\|e_{k}{\|}^{2}. \end{array}$$

Thus, the sufficient descent property (9) holds. Additionally, by applying the Cauchy-Schwartz inequality and using the sufficient descent property, we have:


$$-\|e_{k}\|\|d_{k}\|\leq e_{k}^{\text{T}}d_{k}=-\|e_{k}{\|}^{2},$$

which implies that $\left\|e_{k}\|\leq \|d_{k}\right\|$. □

### Line search analysis

**Lemma 2.** *Suppose that Assumptions (A1) and (A2) hold. Then the following conclusion are satisfied:*

*(1) The line search strategy* (6) *is well-defined.*

*(2) The step-size* $v_{k}$ *satisfies the following relation:*

$$v_{k}\geq \min\left\{\kappa ,\frac{t\|e_{k}{\|}^{2}}{(L+\sigma )\|d_{k}{\|}^{2}}\right\}.$$ (11)

*Proof*: To prove the lemma, we address each part of the statement separately.

Part 1: Suppose, by contradiction, that there exists a positive integer $k_{0}\geq 0$ such that the line search strategy (6) dose not hold for any integer *i* > 0. This means that for all *i* > 0, we obtain:


$$-e(x_{k_{0}}+\kappa t^{i}d_{k_{0}}{)}^{\text{T}}d_{k_{0}}<\sigma \kappa t^{i}\|d_{k_{0}}{\|}^{2}.$$

Given the continuity of e(·), we can take the limit as i→∞, yielding:

$$-e(x_{k_{0}}{)}^{T}d_{k_{0}}\leq 0.$$ (12)

Besides, from the sufficient descent property (6), we know that:


$$-e(x_{k_{0}}{)}^{T}d_{k_{0}}=\|e(x_{k_{0}}){\|}^{2}>0,$$

which contradicts (12). Thus, such a *k*_0_ cannot exist, and we conclude that the line search strategy (6) is well-defined.

Part 2: Assume that $v_{k}\neq \kappa$. In this case, we define ${\bar{v}}_{k}=v_{k}t^{-1}$, which does not satisfy the line search strategy (6), implying that:

$$-e(x_{k}+\bar{v}d_{k}{)}^{\text{T}}d_{k}<\sigma \bar{v}\|d_{k}{\|}^{2}.$$ (13)

Using the sufficient descent property (13) and combining with (9), we obtain the following inequality:


$$\begin{array}{ccc} \|e_{k}{\|}^{2} & = & {\left(e(x_{k}+{\bar{v}}_{k}d_{k})-e_{k}\right)}^{\text{T}}d_{k}-e(x_{k}+{\bar{v}}_{k}d_{k}{)}^{\text{T}}d_{k}\\ & \leq & L\bar{v}\|d_{k}{\|}^{2}+\sigma \bar{v}\|d_{k}{\|}^{2}\\ & = & Lt^{-1}v_{k}\|d_{k}{\|}^{2}+\sigma t^{-1}v_{k}\|d_{k}{\|}^{2}\\ & = & (L+\sigma )t^{-1}v_{k}\|d_{k}{\|}^{2}, \end{array}$$

which is rewritten as:


$$v_{k}\geq \frac{t\|e_{k}{\|}^{2}}{(L+\sigma )\|d_{k}{\|}^{2}}.$$

□

### Global convergence analysis

**Lemma 3.** *Suppose that Assumptions (A1) and (A2) hold. Then, the sequence* $\left\{\|x_{k}-x_{*}\|\right\}$ *is convergent for any* $x_{*}\in E_{*}$. Furthermore, the sequences {*x*_*k*_} *and* {*w*_*k*_} *are bounded, and*

$$\lim_{k\to \infty }v_{k}\|d_{k}\|=0.$$ (14)

*Proof*: The result follows directly from a similar result presented in Lemma 4 of [23]. Therefore, the proof is omitted here for brevity.

**Lemma 4.** *Suppose that Assumptions (A1) and (A2) hold. If* $\|e_{k}\|\geq \varepsilon >0$*, there exists a positive constant N such that, for all* k≥0,

$$\|d_{k}\|\leq N.$$ (15)

*Proof*: From (3), (8), we have the following relationship:


$$\begin{array}{ccc} \left\|u_{k-1}\right\| & = & \left\|e_{k}-e_{k-1}\right\|\\ & \leq & L\|x_{k}-x_{k-1}\|\\ & = & L\|x_{k-1}-\gamma \theta_{k-1}e(w_{k-1})-x_{k-1}\|\\ & \leq & L\gamma \|e(w_{k-1})\|\frac{\left\|e(w_{k-1})\|\|x_{k-1}-w_{k-1}\right\|}{||e(w_{k-1})|{|}^{2}}\\ & = & L\gamma v_{k-1}\|d_{k-1}\|. \end{array}$$

Utilizing this in conjunction with (4), we obtain:

$$\begin{array}{ccc} \left|\beta_{k}^{\text{HCGP}}\right| & \leq & \frac{\left\|e_{k}\|\|u_{k-1}\right\|}{\mu \|d_{k-1}{\|}^{2}}+\chi \frac{\left\|u_{k-1}{\|}^{2}\|e_{k}\|\|d_{k-1}\right\|}{\mu^{2}\|d_{k-1}{\|}^{4}}\\ & \leq & \frac{L\gamma v_{k-1}\|e_{k}\|\|d_{k-1}\|}{\mu \|d_{k-1}{\|}^{2}}+\chi \frac{L^{2}\gamma^{2}v_{k-1}^{2}\|d_{k-1}{\|}^{3}\|e_{k}\|}{\mu^{2}\|d_{k-1}{\|}^{4}}\\ & = & \frac{L\gamma v_{k-1}}{\mu }\frac{\left\|e_{k}\right\|}{\left\|d_{k-1}\right\|}+\frac{\chi L^{2}\gamma^{2}v_{k-1}^{2}}{\mu^{2}}\frac{\left\|e_{k}\right\|}{\left\|d_{k-1}\right\|}\\ & \leq & \left(\frac{L\gamma \kappa }{\mu }+\frac{\chi L^{2}\gamma^{2}\kappa^{2}}{\mu^{2}}\right)\frac{\left\|e_{k}\right\|}{\left\|d_{k-1}\right\|}. \end{array}$$ (16)

Additionally, using Lemma 3, we know that the sequence {*e*_*k*_} is bounded due to the continuity of e(·) and the boundedness of {*x*_*k*_}. That is, there exists a constant *r*>0 such that $\|e_{k}\|\leq r$ holds. From this result, (5), and (16), it follows that


$$\begin{array}{ccc} \left\|d_{k}\right\| & \leq & \left\|e_{k}\|+\left|\beta_{k}^{HCGP}\right|\frac{\left\|e_{k}{\|}^{2}\|d_{k-1}\right\|}{\|e_{k}{\|}^{2}}+\left|\beta_{k}^{HCGP}\right|\|d_{k-1}\right\|\\ & = & \left\|e_{k}\|+2\left|\beta_{k}^{HCGP}\right|\|d_{k-1}\right\|\\ & \leq & \left\|e_{k}\|+2\left(\frac{L\gamma \kappa }{\mu }+\frac{\chi L^{2}\gamma^{2}\kappa^{2}}{\mu^{2}}\right)\frac{\left\|e_{k}\right\|}{\left\|d_{k-1}\right\|}\|d_{k-1}\right\|\\ & = & \left(1+2\left(\frac{L\gamma \kappa }{\mu }+\frac{\chi L^{2}\gamma^{2}\kappa^{2}}{\mu^{2}}\right)\right)\|e_{k}\|\\ & \leq & \left(1+2\left(\frac{L\gamma \kappa }{\mu }+\frac{\chi L^{2}\gamma^{2}\kappa^{2}}{\mu^{2}}\right)\right)r. \end{array}$$

Thus, we have $\|d_{k}\|\leq N$ with $N=\left(1+2\left(\frac{L\gamma \kappa }{\mu }+\frac{\chi L^{2}\gamma^{2}\kappa^{2}}{\mu^{2}}\right)\right)r$. □

**Theorem 1.** *Suppose that Assumptions (A1) and (A2) hold. Then, we have:*

$$\lim_{k\to \infty }\inf\|e_{k}\|=0.$$ (17)

*Proof*: We proceed by assuming the contrary, i.e., (17) does not hold. This implies the existence of a constant ε>0 such that $\|e_{k}\|\geq \varepsilon$ holds for all *k*. Given this assumption, and using the result (10), we immediately deduce the following inequality:

$$\|d_{k}\|\geq \|e_{k}\|\geq \varepsilon .$$ (18)

Next, by multiplying both sides of (11) by $\left\|d_{k}\right\|$, we obtain the following expression:

$$v_{k}\|d_{k}\|\geq \min\left\{\kappa ,\frac{t\|e_{k}{\|}^{2}}{(L+\sigma )\|d_{k}{\|}^{2}}\right\}\|d_{k}\|=\min\left\{\kappa \|d_{k}\|,\frac{t\|e_{k}{\|}^{2}}{\left(L+\sigma )\|d_{k}\right\|}\right\}.$$ (19)

Substituting (15) and (18) into (19), we obtain:


$$v_{k}\|d_{k}\|\geq \min\left\{\kappa \varepsilon ,\frac{t\varepsilon^{2}}{(L+\sigma )N}\right\}.$$

This leads to a contradiction with (14), which shows that our assumption that $\|e_{k}\|\geq \varepsilon$ for all *k* must be false. □

## Numerical experiments

In this section, we compare the performance of the HCGP method with that of two similar methods: HSDY method [31] and DCG method [32]. All experiments are conducted on a Lenovo PC equipped with an Intel(R) Core(TM) i7-12700F processor (2.10GHz), 16GB of RAM, and Windows 11 operation system. For the HCGP method, the following parameters are used:


$$\chi =1,\;\mu =2.4,\;\kappa =1,\;t=0.74,\;\sigma =10^{-4},\;\gamma =1.4,\;\delta =10^{-6}.$$

The parameters for the HSDY and DCG methods are chosen according to the settings specified in the original literature.

In this experiment, we compare the performance of the HCGP, HSDY, and DCG methods on eight benchmark problems. For all methods, the programs are terminated when one of the following criteria is met:


$$(1)\;\|e_{k}\|\leq 10^{-6},\;(2)\;\|d_{k}\|\leq 10^{-6},\;(3)\;\text{NI}>2000,$$

where “NI” is the number of iterations. The dimensions of the benchmark problems is chosen from the set: [1000, 5000, 10000, 50000, 100000]. The initial points for the benchmark problems are selected as follows: $x_{1}=\left(\frac{1}{2},\frac{1}{2^{2}},\ldots ,\frac{1}{2^{n}}\right)$, $x_{2}=\left(0,\frac{1}{n},\ldots ,\frac{n-1}{n}\right)$, $x_{3}=\left(1,\frac{1}{2},\ldots ,\frac{1}{n}\right)$, $x_{4}=\left(\frac{1}{n},\frac{2}{n},\ldots ,\frac{n}{n}\right)$, $x_{5}=\left(\frac{n-1}{n},\frac{n-2}{n},\ldots ,\frac{n-n}{n}\right)$, $x_{6}=(2,2,\ldots ,2)$, $x_{7}=[0,1{]}^{n}$. The specific formulations of the benchmark problems are as follows:

Problem 1:


$$e_{1}(x)=\exp(x_{1})-1,$$

$$e_{i}(x)=\exp(x_{i})+x_{i}-1,\;\text{for}\;i=2,3,\ldots ,n,$$

and $E={\mathbb{R}}_{+}^{n}$.

Problem 2:


$$e_{i}(x)=\exp(x_{i})-1,\;\text{for}\;i=1,2,\ldots ,n,$$

and $E={\mathbb{R}}_{+}^{n}$. Clearly, this problem has a unique solution $x^{*}\;=\;{\left(0,0,\;...\;,0\right)}^{T}$.

Problem 3:


$$e_{i}(x)=2x_{i}-\sin(x_{i}),\;\text{for}\;i=1,2,\ldots ,n,$$

and E=[−2,+∞).

Problem 4;


$$e_{i}(x)=(\exp(x_{i}){)}^{2}+3\sin(x_{i})\cos(x_{i})-1,\;\text{for}\;i=1,2,\ldots ,n,$$

and $E={\mathbb{R}}_{+}^{n}$.

Problem 5:


$$e_{1}(x)=2x_{1}+\sin(x_{1})-1,$$

$$e_{i}(x)=2x_{i-1}+2x_{i}+\sin(x_{i})-1,\;\text{for}\;i=2,3,\ldots ,n-1,$$

$$e_{n}(x)=2x_{n}+\sin(x_{n})-1,$$

and $E={\mathbb{R}}_{+}^{n}$.

Problem 6:


$$e_{i}(x)=\frac{1}{n}\exp(x_{i})-1,\;\text{for}\;i=1,2,\ldots ,n,$$

and $E={\mathbb{R}}_{+}^{n}$.

Problem 7:


$$e_{i}(x)=x_{i}-2\sin(|x_{i}-1|),\;\text{for}\;i=1,2,\ldots ,n,$$

and $E={\mathbb{R}}_{+}^{n}$.

Problem 8:


$$e_{i}(x)=2x_{i}-\sin(|x_{i}|),\;\text{for}\;i=1,2,\ldots ,n,$$

and $E={\mathbb{R}}_{+}^{n}$.

**Fig 1:**
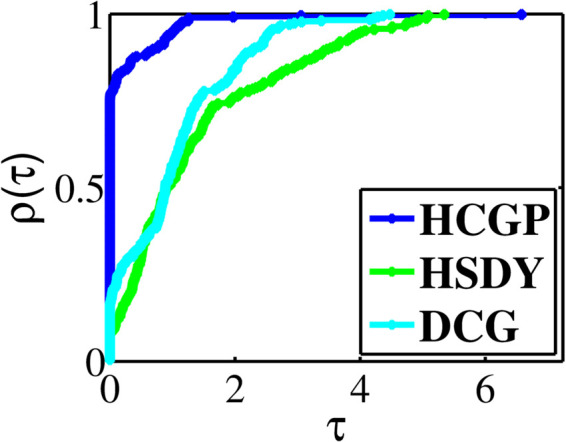
Performance profiles on CPUT.

**Fig 2:**
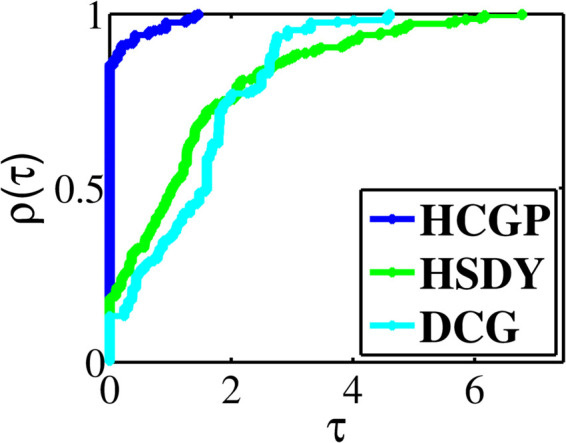
Performance profiles on NFE.

**Fig 3:**
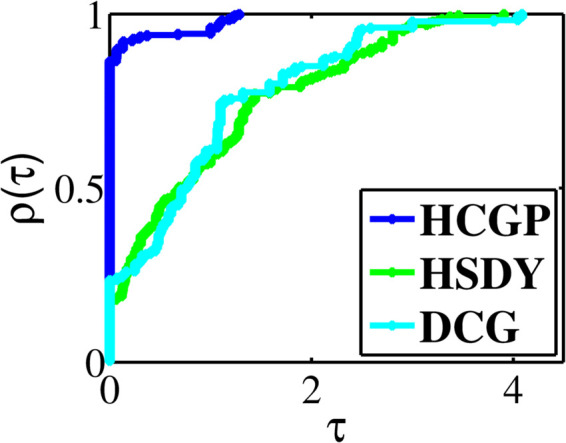
Performance profiles on NI.

The experiment results are summarized in Tables 1–8, where “CPUT” represents the CPU time in second, “NFE” represents the number of function evaluations, “NI” represents the number of iterations, “NORM” represents the norm value of the function at the approximative point, and “Init(*n*)” represents the initial point and the dimension (multiplied by 1000). To provide a visual representation of the computational differences, we utilize the performance profiles introduced by Dolan and Moré [33]. These profiles allow us to assess the relative performance of the methods, where higher curves indicate better numerical efficiency. In Fig 1, the HCGP method demonstrates the lowest CPU time in nearly 75.71% of the benchmark problems, outperforming the HSDY and DCG methods, which achieve this in approximately 8.21%, 16.79% of cases, respectively. Similarly, in Fig 2, the HCGP method consistently shows the fewest number of function evaluations, excelling in almost 85.36% of the benchmark problems. In contrast, the HSDY and DCG methods reach this efficiency in 18.21% and 13.21% of cases, respectively. Lastly, Fig 3 illustrates that the HCGP method also requires the fewest number of iterations in 86.43% of the benchmark problems, while the HSDY and DCG methods perform better in only in 17.86% and 23.57% of cases, respectively.

**Table 1:** Numerical results for Problem 1.

	HCGP	HSDY	DCG
Inti(*n*)	CPUT/NFE/NI/NORM	CPUT/NFE/NI/NORM	CPUT/NFE/NI/NORM
*x*_1_(1)	3.02e-02/15/3/1.09e-15	3.63e-03/87/13/3.30e-07	3.67e-03/39/6/1.88e-15
*x*_2_(1)	2.26e-03/32/6/9.38e-07	4.23e-03/99/14/0.00e+00	1.87e-03/112/15/8.03e-08
*x*_3_(1)	3.96e-04/11/2/0.00e+00	1.55e-03/79/13/1.99e-07	2.33e-04/13/2/0.00e+00
*x*_4_(1)	6.73e-04/32/6/9.46e-07	1.65e-03/91/13/3.51e-07	1.86e-03/112/15/8.16e-08
*x*_5_(1)	6.62e-04/32/6/9.47e-07	3.56e-03/201/29/7.44e-07	1.85e-03/112/15/8.05e-08
*x*_6_(1)	1.87e-04/8/1/0.00e+00	2.06e-04/10/1/0.00e+00	2.27e-04/8/1/0.00e+00
*x*_7_(1)	8.68e-04/32/6/7.60e-07	2.45e-03/103/16/7.98e-07	1.96e-03/112/15/7.72e-08
*x*_1_(5)	3.37e-02/4/1/0.00e+00	3.03e-03/17/7/8.81e-08	3.52e-04/4/1/0.00e+00
*x*_2_(5)	4.79e-03/37/7/9.35e-09	1.88e-02/185/27/4.05e-07	9.97e-03/112/15/1.81e-07
*x*_3_(5)	1.15e-03/11/2/0.00e+00	1.15e-02/113/18/2.62e-07	1.07e-03/13/2/0.00e+00
*x*_4_(5)	3.59e-03/37/7/9.36e-09	1.88e-02/200/29/7.05e-07	9.41e-03/112/15/1.81e-07
*x*_5_(5)	3.57e-03/37/7/9.36e-09	1.38e-02/145/21/1.27e-07	9.60e-03/112/15/1.81e-07
*x*_6_(5)	7.31e-04/8/1/0.00e+00	9.13e-04/10/1/0.00e+00	7.01e-04/8/1/0.00e+00
*x*_7_(5)	3.96e-03/37/7/5.37e-09	7.14e-03/70/11/2.37e-07	9.60e-03/112/15/1.85e-07
*x*_1_(10)	7.37e-04/4/1/0.00e+00	5.22e-03/17/7/8.81e-08	6.85e-04/4/1/0.00e+00
*x*_2_(10)	6.07e-03/37/7/1.32e-08	1.87e-02/124/19/7.40e-07	1.55e-02/112/15/2.56e-07
*x*_3_(10)	1.68e-03/11/2/0.00e+00	1.55e-02/87/15/0.00e+00	1.71e-03/13/2/0.00e+00
*x*_4_(10)	6.01e-03/37/7/1.32e-08	1.51e-02/92/14/6.04e-07	1.46e-02/112/15/2.56e-07
*x*_5_(10)	5.75e-03/37/7/1.32e-08	2.45e-02/153/22/1.11e-07	1.61e-02/112/15/2.56e-07
*x*_6_(10)	1.19e-03/8/1/0.00e+00	1.50e-03/10/1/0.00e+00	1.04e-03/8/1/0.00e+00
*x*_7_(10)	6.44e-03/37/7/7.92e-09	1.03e-02/60/10/8.17e-07	1.70e-02/112/15/2.57e-07
*x*_1_(50)	3.03e-03/4/1/0.00e+00	1.35e-02/17/7/8.81e-08	2.45e-03/4/1/0.00e+00
*x*_2_(50)	2.32e-02/37/7/2.96e-08	7.63e-02/125/19/3.14e-07	5.72e-02/112/15/5.72e-07
*x*_3_(50)	6.79e-03/11/2/0.00e+00	4.38e-02/71/12/1.93e-07	6.45e-03/13/2/0.00e+00
*x*_4_(50)	2.28e-02/37/7/2.96e-08	3.28e-02/57/8/0.00e+00	5.57e-02/112/15/5.73e-07
*x*_5_(50)	2.20e-02/37/7/2.96e-08	6.24e-02/105/16/2.44e-07	5.80e-02/112/15/5.72e-07
*x*_6_(50)	4.80e-03/8/1/0.00e+00	5.24e-03/10/1/0.00e+00	4.62e-03/8/1/0.00e+00
*x*_7_(50)	2.46e-02/37/7/2.65e-08	2.84e-02/43/6/1.09e-08	5.77e-02/112/15/5.69e-07
*x*_1_(100)	6.48e-03/4/1/0.00e+00	2.73e-02/17/7/8.81e-08	4.98e-03/4/1/0.00e+00
*x*_2_(100)	4.17e-02/37/7/4.18e-08	1.01e-01/89/14/2.80e-07	1.09e-01/112/15/8.09e-07
*x*_3_(100)	1.31e-02/11/2/0.00e+00	8.69e-02/71/12/2.51e-07	1.40e-02/13/2/0.00e+00
*x*_4_(100)	4.24e-02/37/7/4.18e-08	1.60e-01/151/22/1.07e-07	1.07e-01/112/15/8.10e-07
*x*_5_(100)	4.36e-02/37/7/4.18e-08	1.21e-01/112/17/4.17e-07	1.03e-01/112/15/8.09e-07
*x*_6_(100)	8.36e-03/8/1/0.00e+00	1.03e-02/10/1/0.00e+00	8.62e-03/8/1/0.00e+00
*x*_7_(100)	5.03e-02/37/7/3.86e-08	1.57e-01/120/18/5.44e-07	1.10e-01/112/15/8.07e-07

**Table 2:** Numerical results for Problem 2.

	HCGP	HSDY	DCG
Inti(*n*)	CPUT/NFE/NI/NORM	CPUT/NFE/NI/NORM	CPUT/NFE/NI/NORM
*x*_1_(1)	1.76e-04/7/2/2.22e-16	4.60e-04/19/8/6.46e-07	7.26e-04/65/12/4.91e-07
*x*_2_(1)	6.42e-04/33/10/1.41e-07	3.25e-03/269/24/3.61e-08	1.18e-03/89/16/8.72e-07
*x*_3_(1)	1.71e-04/9/2/0.00e+00	5.15e-04/33/6/0.00e+00	1.14e-03/89/16/3.68e-07
*x*_4_(1)	5.85e-04/30/9/9.94e-07	1.73e-03/123/18/4.21e-07	1.16e-03/89/16/8.80e-07
*x*_5_(1)	6.46e-04/33/10/1.41e-07	3.16e-03/269/24/3.61e-08	1.14e-03/89/16/8.72e-07
*x*_6_(1)	1.37e-04/7/1/0.00e+00	1.45e-04/9/1/0.00e+00	1.09e-04/7/1/0.00e+00
*x*_7_(1)	6.50e-04/30/9/3.49e-07	2.66e-03/209/22/6.66e-08	1.19e-03/89/16/8.48e-07
*x*_1_(5)	5.61e-04/4/1/0.00e+00	2.85e-03/17/7/8.81e-08	2.98e-04/4/1/0.00e+00
*x*_2_(5)	3.09e-03/33/10/3.22e-07	3.21e-02/560/37/4.81e-08	7.01e-03/100/18/2.07e-07
*x*_3_(5)	8.01e-04/9/2/0.00e+00	4.84e-03/50/13/5.04e-07	6.19e-03/89/16/3.68e-07
*x*_4_(5)	3.22e-03/33/10/3.19e-07	2.81e-02/484/37/6.98e-07	6.77e-03/100/18/2.08e-07
*x*_5_(5)	3.13e-03/33/10/3.22e-07	3.08e-02/560/37/4.81e-08	7.49e-03/100/18/2.07e-07
*x*_6_(5)	5.22e-04/7/1/0.00e+00	6.73e-04/9/1/0.00e+00	5.20e-04/7/1/0.00e+00
*x*_7_(5)	3.37e-03/33/10/1.29e-07	4.66e-02/856/51/5.01e-07	7.16e-03/100/18/2.07e-07
*x*_1_(10)	6.80e-04/4/1/0.00e+00	3.31e-03/17/7/8.81e-08	4.27e-04/4/1/0.00e+00
*x*_2_(10)	5.77e-03/33/10/4.50e-07	7.39e-02/956/54/3.86e-07	1.18e-02/100/18/2.93e-07
*x*_3_(10)	1.11e-03/9/2/0.00e+00	7.93e-03/48/13/6.83e-07	9.29e-03/89/16/3.68e-07
*x*_4_(10)	5.66e-03/33/10/4.49e-07	8.32e-02/1011/60/4.59e-07	1.07e-02/100/18/2.94e-07
*x*_5_(10)	5.37e-03/33/10/4.50e-07	7.61e-02/956/54/3.86e-07	1.13e-02/100/18/2.93e-07
*x*_6_(10)	6.98e-04/7/1/0.00e+00	8.98e-04/9/1/0.00e+00	8.35e-04/7/1/0.00e+00
*x*_7_(10)	5.98e-03/33/10/2.81e-07	8.52e-02/975/57/3.00e-07	1.04e-02/100/18/2.93e-07
*x*_1_(50)	2.47e-03/4/1/0.00e+00	1.09e-02/17/7/8.81e-08	1.03e-03/4/1/0.00e+00
*x*_2_(50)	2.07e-02/36/11/8.40e-08	4.73e-01/1845/86/0.00e+00	3.60e-02/100/18/6.56e-07
*x*_3_(50)	4.63e-03/9/2/0.00e+00	2.77e-02/48/13/9.22e-07	2.99e-02/89/16/3.68e-07
*x*_4_(50)	1.97e-02/36/11/8.39e-08	5.29e-01/2079/97/8.13e-07	3.48e-02/100/18/6.56e-07
*x*_5_(50)	2.08e-02/36/11/8.40e-08	4.71e-01/1845/86/0.00e+00	3.47e-02/100/18/6.56e-07
*x*_6_(50)	2.96e-03/7/1/0.00e+00	3.37e-03/9/1/0.00e+00	2.20e-03/7/1/0.00e+00
*x*_7_(50)	2.21e-02/33/10/9.31e-07	4.13e-01/1580/77/9.39e-07	3.74e-02/100/18/6.53e-07
*x*_1_(100)	4.09e-03/4/1/0.00e+00	1.63e-02/17/7/8.81e-08	2.68e-03/4/1/0.00e+00
*x*_2_(100)	3.33e-02/36/11/1.19e-07	1.11e+00/2583/114/8.49e-07	5.74e-02/100/18/9.28e-07
*x*_3_(100)	7.02e-03/9/2/0.00e+00	5.59e-02/58/15/4.40e-07	5.13e-02/89/16/3.68e-07
*x*_4_(100)	3.42e-02/36/11/1.19e-07	1.05e+00/2460/108/0.00e+00	5.72e-02/100/18/9.28e-07
*x*_5_(100)	3.23e-02/36/11/1.19e-07	1.10e+00/2583/114/8.49e-07	5.82e-02/100/18/9.28e-07
*x*_6_(100)	4.61e-03/7/1/0.00e+00	6.10e-03/9/1/0.00e+00	3.65e-03/7/1/0.00e+00
*x*_7_(100)	4.01e-02/36/11/1.11e-07	1.63e+00/3956/165/2.71e-07	6.20e-02/100/18/9.26e-07

**Table 3:** Numerical results for Problem 3.

	HCGP	HSDY	DCG
Inti(*n*)	CPUT/NFE/NI/NORM	CPUT/NFE/NI/NORM	CPUT/NFE/NI/NORM
*x*_1_(1)	4.43e-03/22/7/8.51e-07	1.33e-03/59/17/1.81e-07	1.13e-03/77/15/8.83e-07
*x*_2_(1)	6.38e-04/31/10/3.14e-07	1.72e-03/90/25/1.79e-07	1.40e-03/107/21/3.77e-07
*x*_3_(1)	5.62e-04/28/9/1.74e-07	1.37e-03/72/20/1.85e-07	1.24e-03/98/19/4.73e-07
*x*_4_(1)	6.27e-04/31/10/3.15e-07	9.03e-04/42/14/9.29e-07	1.40e-03/107/21/3.30e-07
*x*_5_(1)	6.23e-04/31/10/3.14e-07	1.71e-03/90/25/1.91e-07	1.41e-03/107/21/3.77e-07
*x*_6_(1)	4.57e-04/23/7/4.21e-08	6.56e-04/26/11/1.93e-09	4.62e-04/29/9/7.39e-07
*x*_7_(1)	6.49e-04/31/10/2.94e-07	1.50e-03/76/22/9.81e-07	1.41e-03/107/21/5.92e-07
*x*_1_(5)	2.12e-03/16/5/6.65e-08	1.69e-03/12/5/5.50e-08	2.29e-03/22/7/2.51e-07
*x*_2_(5)	4.00e-03/31/10/7.02e-07	8.53e-03/66/20/9.18e-07	7.23e-03/107/21/7.97e-07
*x*_3_(5)	3.02e-03/28/9/1.74e-07	5.14e-03/52/16/5.24e-07	6.79e-03/98/19/4.74e-07
*x*_4_(5)	3.02e-03/31/10/7.03e-07	6.00e-03/59/18/6.78e-07	7.13e-03/107/21/7.76e-07
*x*_5_(5)	3.56e-03/31/10/7.02e-07	7.01e-03/66/20/9.18e-07	7.34e-03/107/21/7.97e-07
*x*_6_(5)	6.45e-03/68/21/2.19e-07	3.38e-03/26/11/4.31e-09	2.70e-03/32/10/1.98e-07
*x*_7_(5)	3.20e-03/31/10/6.96e-07	8.11e-03/82/24/9.74e-07	7.38e-03/107/21/8.43e-07
*x*_1_(10)	2.44e-03/16/5/6.65e-08	1.96e-03/12/5/5.50e-08	2.75e-03/22/7/2.51e-07
*x*_2_(10)	5.56e-03/31/10/9.94e-07	1.05e-02/70/21/3.88e-07	1.31e-02/112/22/9.30e-07
*x*_3_(10)	4.29e-03/28/9/1.74e-07	9.98e-03/55/16/9.07e-07	1.06e-02/98/19/4.74e-07
*x*_4_(10)	5.04e-03/31/10/9.94e-07	1.67e-02/103/30/7.55e-07	1.39e-02/112/22/9.17e-07
*x*_5_(10)	4.32e-03/31/10/9.94e-07	1.27e-02/70/21/3.88e-07	1.11e-02/112/22/9.30e-07
*x*_6_(10)	4.10e-03/24/8/5.91e-07	5.54e-03/26/11/6.10e-09	4.19e-03/32/10/2.80e-07
*x*_7_(10)	5.01e-03/31/10/9.79e-07	1.04e-02/74/22/9.09e-07	1.20e-02/118/23/2.07e-07
*x*_1_(50)	7.32e-03/16/5/6.65e-08	6.46e-03/12/5/5.50e-08	6.79e-03/22/7/2.51e-07
*x*_2_(50)	2.05e-02/34/11/3.19e-07	3.77e-02/68/20/7.88e-07	4.21e-02/118/23/3.92e-07
*x*_3_(50)	1.73e-02/28/9/1.74e-07	3.93e-02/70/20/8.56e-07	3.43e-02/98/19/4.74e-07
*x*_4_(50)	1.83e-02/34/11/3.19e-07	5.01e-02/91/26/5.65e-07	4.26e-02/118/23/3.91e-07
*x*_5_(50)	2.03e-02/34/11/3.19e-07	3.80e-02/68/20/7.88e-07	4.34e-02/118/23/3.92e-07
*x*_6_(50)	1.25e-02/23/7/2.97e-07	1.84e-02/26/11/1.36e-08	1.28e-02/32/10/6.27e-07
*x*_7_(50)	1.92e-02/34/11/3.17e-07	5.36e-02/99/28/2.02e-07	4.20e-02/118/23/4.71e-07
*x*_1_(100)	1.05e-02/16/5/6.65e-08	1.18e-02/12/5/5.50e-08	1.19e-02/22/7/2.51e-07
*x*_2_(100)	3.20e-02/34/11/4.51e-07	7.68e-02/82/24/6.26e-07	6.91e-02/118/23/5.55e-07
*x*_3_(100)	2.70e-02/28/9/1.74e-07	5.59e-02/54/16/8.66e-07	5.77e-02/98/19/4.74e-07
*x*_4_(100)	3.22e-02/34/11/4.51e-07	8.97e-02/89/26/6.94e-07	6.73e-02/118/23/5.54e-07
*x*_5_(100)	2.96e-02/34/11/4.51e-07	7.90e-02/82/24/6.25e-07	6.86e-02/118/23/5.55e-07
*x*_6_(100)	2.40e-02/26/9/7.48e-07	3.35e-02/26/11/1.93e-08	2.23e-02/32/10/8.87e-07
*x*_7_(100)	2.98e-02/34/11/4.50e-07	5.32e-02/54/16/9.87e-07	6.70e-02/118/23/6.11e-07

**Table 4:** Numerical results for Problem 4.

	HCGP	HSDY	DCG
Inti(*n*)	CPUT/NFE/NI/NORM	CPUT/NFE/NI/NORM	CPUT/NFE/NI/NORM
*x*_1_(1)	3.58e-04/17/2/2.22e-16	1.52e-03/104/10/2.61e-15	3.73e-03/274/28/6.06e-07
*x*_2_(1)	1.35e-03/67/8/6.33e-07	4.51e-02/745/32/0.00e+00	7.13e-03/439/44/6.68e-07
*x*_3_(1)	3.00e-04/14/2/0.00e+00	2.50e-03/151/13/0.00e+00	5.36e-03/340/34/7.89e-07
*x*_4_(1)	1.31e-03/67/8/5.89e-07	3.50e-02/623/30/0.00e+00	7.22e-03/439/44/9.85e-07
*x*_5_(1)	1.30e-03/67/8/6.33e-07	3.88e-02/690/30/0.00e+00	7.10e-03/439/44/6.68e-07
*x*_6_(1)	1.73e-04/4/1/0.00e+00	1.67e-04/4/1/0.00e+00	2.93e-04/10/1/0.00e+00
*x*_7_(1)	1.89e-03/83/10/1.30e-08	5.44e-03/304/25/2.79e-08	8.00e-03/439/44/7.06e-07
*x*_1_(5)	6.92e-04/3/1/0.00e+00	5.29e-04/3/1/0.00e+00	4.64e-04/3/1/0.00e+00
*x*_2_(5)	8.58e-03/75/9/1.36e-08	8.41e-02/401/16/0.00e+00	3.32e-02/459/46/7.22e-07
*x*_3_(5)	1.12e-03/14/2/0.00e+00	4.21e-03/62/5/0.00e+00	2.49e-02/340/34/7.07e-07
*x*_4_(5)	5.86e-03/75/9/1.34e-08	9.54e-02/551/24/0.00e+00	3.41e-02/459/46/9.27e-07
*x*_5_(5)	5.48e-03/75/9/1.36e-08	8.15e-02/403/17/0.00e+00	3.33e-02/459/46/7.22e-07
*x*_6_(5)	5.38e-04/4/1/0.00e+00	5.48e-04/4/1/0.00e+00	9.57e-04/10/1/0.00e+00
*x*_7_(5)	8.08e-03/83/10/2.48e-08	8.44e-02/379/14/0.00e+00	3.50e-02/459/46/9.65e-07
*x*_1_(10)	5.98e-04/3/1/0.00e+00	4.90e-04/3/1/0.00e+00	2.97e-04/3/1/0.00e+00
*x*_2_(10)	7.18e-03/75/9/2.06e-08	2.04e-01/2045/86/0.00e+00	4.09e-02/469/47/8.59e-07
*x*_3_(10)	1.47e-03/14/2/0.00e+00	6.82e-03/62/5/0.00e+00	2.86e-02/330/33/9.66e-07
*x*_4_(10)	8.61e-03/75/9/2.04e-08	5.50e-02/574/22/0.00e+00	4.15e-02/469/47/8.99e-07
*x*_5_(10)	7.18e-03/75/9/2.06e-08	1.89e-01/1942/83/0.00e+00	4.23e-02/469/47/8.59e-07
*x*_6_(10)	6.38e-04/4/1/0.00e+00	7.18e-04/4/1/0.00e+00	1.25e-03/10/1/0.00e+00
*x*_7_(10)	1.09e-02/83/10/1.53e-08	1.38e-01/1829/52/6.44e-07	4.45e-02/469/47/9.24e-07
*x*_1_(50)	2.20e-03/3/1/0.00e+00	1.74e-03/3/1/0.00e+00	1.06e-03/3/1/0.00e+00
*x*_2_(50)	3.09e-02/75/9/4.59e-08	4.75e-01/1080/39/0.00e+00	1.73e-01/489/49/8.86e-07
*x*_3_(50)	5.63e-03/14/2/0.00e+00	2.36e-02/62/5/0.00e+00	1.16e-01/330/33/8.92e-07
*x*_4_(50)	3.25e-02/75/9/4.59e-08	5.15e-01/1623/56/0.00e+00	1.71e-01/489/49/8.91e-07
*x*_5_(50)	3.08e-02/75/9/4.59e-08	3.88e-01/1299/48/0.00e+00	1.83e-01/489/49/8.86e-07
*x*_6_(50)	2.41e-03/4/1/0.00e+00	2.54e-03/4/1/0.00e+00	5.15e-03/10/1/0.00e+00
*x*_7_(50)	3.84e-02/75/9/3.43e-08	4.80e-01/1133/42/0.00e+00	1.85e-01/489/49/7.60e-07
*x*_1_(100)	3.29e-03/3/1/0.00e+00	3.17e-03/3/1/0.00e+00	2.03e-03/3/1/0.00e+00
*x*_2_(100)	5.46e-02/75/9/6.50e-08	6.33e-01/1287/48/0.00e+00	2.99e-01/499/50/8.27e-07
*x*_3_(100)	1.03e-02/14/2/0.00e+00	3.95e-02/62/5/0.00e+00	1.92e-01/330/33/8.82e-07
*x*_4_(100)	5.39e-02/75/9/6.49e-08	6.00e-01/1196/46/0.00e+00	2.98e-01/499/50/8.22e-07
*x*_5_(100)	5.26e-02/75/9/6.50e-08	8.90e-01/1146/41/0.00e+00	3.02e-01/499/50/8.27e-07
*x*_6_(100)	1.02e-02/11/1/0.00e+00	5.59e-03/4/1/0.00e+00	9.47e-03/10/1/0.00e+00
*x*_7_(100)	6.26e-02/75/9/5.60e-08	5.27e-01/1053/38/0.00e+00	3.05e-01/489/49/9.06e-07

**Table 5:** Numerical results for Problem 5.

	HCGP	HSDY	DCG
Inti(*n*)	CPUT/NFE/NI/NORM	CPUT/NFE/NI/NORM	CPUT/NFE/NI/NORM
*x*_1_(1)	4.43e-03/195/25/5.47e-07	7.85e-03/375/33/8.48e-07	5.83e-03/345/35/6.55e-07
*x*_2_(1)	3.11e-03/161/20/5.15e-07	6.77e-03/390/34/6.69e-07	6.33e-03/375/38/8.72e-07
*x*_3_(1)	3.11e-03/160/20/9.70e-07	7.74e-03/446/39/7.85e-07	5.74e-03/334/34/6.13e-07
*x*_4_(1)	3.12e-03/161/20/5.14e-07	6.77e-03/389/34/7.03e-07	6.36e-03/375/38/7.60e-07
*x*_5_(1)	3.14e-03/161/20/7.77e-07	7.27e-03/424/37/6.56e-07	6.36e-03/373/38/6.93e-07
*x*_6_(1)	3.84e-03/195/25/6.46e-07	8.58e-03/488/43/9.75e-07	5.50e-03/321/33/8.66e-07
*x*_7_(1)	1.37e-02/692/95/6.66e-07	9.15e-03/517/45/6.39e-07	6.69e-03/388/39/7.78e-07
*x*_1_(5)	3.12e-02/251/33/4.69e-07	3.76e-02/395/35/9.88e-07	3.36e-02/360/37/7.14e-07
*x*_2_(5)	1.89e-02/182/23/7.24e-07	3.90e-02/418/37/9.69e-07	3.74e-02/394/40/5.62e-07
*x*_3_(5)	2.09e-02/195/25/9.97e-07	3.69e-02/375/33/8.37e-07	3.51e-02/364/37/9.12e-07
*x*_4_(5)	1.89e-02/182/23/7.24e-07	4.28e-02/444/39/7.57e-07	3.70e-02/394/40/5.61e-07
*x*_5_(5)	1.98e-02/189/24/8.22e-07	3.67e-02/399/35/2.64e-07	3.58e-02/381/39/8.68e-07
*x*_6_(5)	1.94e-02/181/23/9.86e-07	4.42e-02/475/42/7.64e-07	3.37e-02/354/37/7.94e-07
*x*_7_(5)	7.12e-02/679/93/8.92e-07	5.02e-02/530/46/4.77e-07	3.92e-02/408/41/6.89e-07
*x*_1_(10)	3.47e-02/202/26/7.38e-07	6.12e-02/400/35/9.84e-07	5.57e-02/352/36/8.77e-07
*x*_2_(10)	3.42e-02/196/25/9.18e-07	7.11e-02/457/40/2.94e-07	6.10e-02/404/41/5.66e-07
*x*_3_(10)	3.69e-02/210/27/7.58e-07	6.82e-02/434/38/9.80e-07	5.42e-02/364/37/7.21e-07
*x*_4_(10)	3.47e-02/196/25/9.18e-07	6.23e-02/409/36/9.09e-07	6.12e-02/404/41/5.78e-07
*x*_5_(10)	3.69e-02/211/27/4.63e-07	6.62e-02/434/38/6.63e-07	5.71e-02/377/40/7.45e-07
*x*_6_(10)	2.94e-02/175/22/6.81e-07	7.08e-02/462/41/8.27e-07	5.42e-02/359/37/7.93e-07
*x*_7_(10)	1.18e-01/659/90/6.89e-07	6.54e-02/434/38/9.61e-07	6.22e-02/418/42/7.31e-07
*x*_1_(50)	1.10e-01/168/21/6.75e-07	2.42e-01/411/36/5.50e-07	2.16e-01/368/38/7.69e-07
*x*_2_(50)	1.28e-01/197/25/6.51e-07	2.00e-01/339/30/8.51e-07	2.44e-01/420/43/6.66e-07
*x*_3_(50)	1.16e-01/169/21/8.61e-07	2.62e-01/446/39/4.91e-07	2.07e-01/363/37/9.45e-07
*x*_4_(50)	1.28e-01/197/25/6.51e-07	2.04e-01/361/32/9.75e-07	2.37e-01/420/43/6.66e-07
*x*_5_(50)	1.34e-01/211/27/7.00e-07	2.43e-01/429/38/9.65e-07	2.31e-01/410/42/5.62e-07
*x*_6_(50)	1.32e-01/203/26/5.24e-07	2.97e-01/518/46/3.37e-07	2.02e-01/357/37/8.11e-07
*x*_7_(50)	4.44e-01/667/91/8.27e-07	3.33e-01/552/48/6.96e-07	2.58e-01/428/43/9.35e-07
*x*_1_(100)	2.38e-01/189/24/6.69e-07	4.94e-01/410/36/6.94e-07	4.56e-01/405/42/7.79e-07
*x*_2_(100)	2.86e-01/232/30/8.47e-07	5.49e-01/465/41/3.50e-07	4.43e-01/401/41/8.65e-07
*x*_3_(100)	2.19e-01/176/22/6.06e-07	4.92e-01/398/35/8.32e-07	4.30e-01/391/40/8.40e-07
*x*_4_(100)	3.02e-01/232/30/8.47e-07	5.26e-01/442/39/8.29e-07	4.40e-01/401/41/8.66e-07
*x*_5_(100)	3.13e-01/239/31/8.94e-07	4.65e-01/384/34/6.89e-07	4.62e-01/422/43/6.36e-07
*x*_6_(100)	3.02e-01/237/31/8.81e-07	5.30e-01/439/39/5.80e-07	3.89e-01/357/37/9.80e-07
*x*_7_(100)	8.61e-01/682/93/6.11e-07	6.61e-01/549/48/8.81e-07	4.73e-01/438/44/9.63e-07

**Table 6:** Numerical results for Problem 6.

	HCGP	HSDY	DCG
Inti(*n*)	CPUT/NFE/NI/NORM	CPUT/NFE/NI/NORM	CPUT/NFE/NI/NORM
*x*_1_(1)	1.38e-03/45/18/5.12e-07	1.30e-03/37/18/7.23e-07	2.04e-03/106/22/4.85e-07
*x*_2_(1)	1.20e-03/42/16/2.87e-07	1.27e-03/37/18/5.55e-07	2.21e-03/118/24/4.82e-07
*x*_3_(1)	1.39e-03/48/19/1.33e-07	1.27e-03/37/18/7.16e-07	2.17e-03/116/24/5.68e-07
*x*_4_(1)	1.19e-03/42/16/2.92e-07	1.27e-03/37/18/5.55e-07	2.20e-03/118/24/5.50e-07
*x*_5_(1)	1.18e-03/42/16/2.87e-07	1.26e-03/37/18/5.55e-07	2.19e-03/118/24/4.82e-07
*x*_6_(1)	8.54e-04/29/12/1.30e-07	1.20e-03/35/17/2.34e-07	9.25e-04/40/15/8.40e-07
*x*_7_(1)	1.18e-03/42/16/2.12e-07	1.27e-03/37/18/5.01e-07	2.80e-03/150/31/3.69e-07
*x*_1_(5)	7.59e-03/47/19/1.91e-07	8.36e-03/41/20/5.35e-07	1.50e-02/155/27/3.90e-07
*x*_2_(5)	5.64e-03/46/18/2.94e-07	6.35e-03/42/20/1.78e-07	1.10e-02/131/26/7.24e-07
*x*_3_(5)	7.35e-03/54/22/3.80e-07	6.90e-03/41/20/5.33e-07	1.25e-02/150/30/3.51e-07
*x*_4_(5)	5.61e-03/46/18/2.94e-07	7.05e-03/42/20/1.77e-07	1.07e-02/131/26/7.30e-07
*x*_5_(5)	5.87e-03/46/18/2.94e-07	6.25e-03/42/20/1.78e-07	1.03e-02/131/26/7.24e-07
*x*_6_(5)	3.99e-03/31/13/6.18e-07	5.67e-03/37/18/9.68e-07	4.45e-03/43/16/1.36e-07
*x*_7_(5)	5.82e-03/46/18/2.92e-07	6.24e-03/42/20/1.23e-07	1.12e-02/136/27/3.03e-07
*x*_1_(10)	7.16e-03/46/19/6.60e-07	9.10e-03/43/21/4.56e-07	1.56e-02/167/29/2.04e-07
*x*_2_(10)	6.20e-03/42/16/2.75e-07	9.51e-03/48/22/8.04e-07	1.65e-02/167/31/3.05e-07
*x*_3_(10)	8.36e-03/56/23/2.60e-07	8.60e-03/43/21/4.55e-07	1.52e-02/157/30/5.91e-07
*x*_4_(10)	6.46e-03/42/16/2.76e-07	1.12e-02/48/22/8.05e-07	1.59e-02/166/31/9.56e-07
*x*_5_(10)	6.35e-03/42/16/2.75e-07	9.53e-03/48/22/8.04e-07	1.62e-02/167/31/3.05e-07
*x*_6_(10)	4.90e-03/31/13/7.15e-07	8.12e-03/39/19/6.60e-07	5.80e-03/47/18/2.44e-07
*x*_7_(10)	6.50e-03/42/16/2.71e-07	9.63e-03/48/22/6.83e-07	1.61e-02/166/31/1.91e-07
*x*_1_(50)	3.08e-02/44/18/8.36e-07	3.92e-02/47/23/4.30e-07	7.52e-02/212/33/9.20e-07
*x*_2_(50)	3.21e-02/48/19/7.77e-07	3.92e-02/46/22/4.76e-07	5.90e-02/148/29/5.81e-07
*x*_3_(50)	3.29e-02/47/19/9.87e-07	3.92e-02/47/23/4.30e-07	2.14e-01/631/71/3.08e-07
*x*_4_(50)	3.26e-02/48/19/7.77e-07	3.95e-02/46/22/4.76e-07	5.70e-02/148/29/5.84e-07
*x*_5_(50)	3.24e-02/48/19/7.77e-07	3.75e-02/46/22/4.76e-07	5.90e-02/148/29/5.81e-07
*x*_6_(50)	2.24e-02/31/13/2.29e-07	3.89e-02/43/21/7.02e-07	2.56e-02/48/19/4.61e-07
*x*_7_(50)	3.33e-02/48/19/7.75e-07	3.98e-02/46/22/4.65e-07	5.68e-02/148/29/6.96e-07
*x*_1_(100)	5.58e-02/48/20/4.92e-07	7.71e-02/49/24/3.40e-07	8.17e-02/120/25/4.77e-07
*x*_2_(100)	5.72e-02/53/21/1.72e-07	7.98e-02/57/25/5.23e-07	8.05e-02/121/25/5.27e-07
*x*_3_(100)	6.15e-02/54/22/1.54e-07	7.90e-02/49/24/3.40e-07	1.14e-01/181/31/2.99e-07
*x*_4_(100)	5.84e-02/53/21/1.72e-07	8.35e-02/57/25/5.23e-07	7.95e-02/121/25/5.27e-07
*x*_5_(100)	5.91e-02/53/21/1.72e-07	8.41e-02/57/25/5.23e-07	8.17e-02/121/25/5.27e-07
*x*_6_(100)	4.45e-02/38/16/1.41e-07	7.26e-02/45/22/5.38e-07	4.12e-02/49/19/6.59e-07
*x*_7_(100)	6.08e-02/53/21/1.72e-07	7.99e-02/57/25/5.81e-07	7.88e-02/121/25/5.19e-07

**Table 7:** Numerical results for Problem 7.

	HCGP	HSDY	DCG
Inti(*n*)	CPUT/NFE/NI/NORM	CPUT/NFE/NI/NORM	CPUT/NFE/NI/NORM
*x*_1_(1)	2.85e-03/121/20/4.13e-07	1.92e-03/142/17/7.64e-07	2.33e-03/190/24/1.84e-07
*x*_2_(1)	1.94e-03/127/21/5.33e-07	2.94e-03/225/26/3.35e-07	2.10e-03/172/21/3.16e-07
*x*_3_(1)	1.87e-03/121/20/5.05e-07	2.69e-03/205/24/7.92e-07	2.42e-03/200/25/2.59e-07
*x*_4_(1)	1.94e-03/127/21/5.33e-07	1.93e-03/145/17/1.42e-07	2.09e-03/172/21/3.11e-07
*x*_5_(1)	1.95e-03/127/21/5.33e-07	2.98e-03/225/26/3.35e-07	2.11e-03/172/21/3.16e-07
*x*_6_(1)	1.16e-03/71/13/7.39e-07	5.26e-04/29/6/2.02e-07	2.17e-03/158/32/7.44e-07
*x*_7_(1)	1.99e-03/127/21/5.00e-07	2.29e-03/172/20/2.12e-07	2.18e-03/172/21/3.37e-07
*x*_1_(5)	1.28e-02/115/19/9.24e-07	1.44e-02/182/21/2.01e-07	1.09e-02/155/20/4.05e-07
*x*_2_(5)	1.24e-02/133/22/8.27e-07	1.39e-02/200/23/1.60e-07	1.25e-02/172/21/7.02e-07
*x*_3_(5)	9.32e-03/121/20/7.45e-07	1.83e-02/225/26/5.21e-07	1.48e-02/223/28/5.20e-07
*x*_4_(5)	1.19e-02/133/22/8.27e-07	1.65e-02/225/26/6.97e-07	1.18e-02/172/21/7.00e-07
*x*_5_(5)	1.06e-02/133/22/8.27e-07	1.44e-02/200/23/1.60e-07	1.21e-02/172/21/7.02e-07
*x*_6_(5)	6.29e-03/77/14/3.50e-07	2.80e-03/29/6/4.53e-07	1.27e-02/163/33/9.72e-07
*x*_7_(5)	1.16e-02/133/22/8.13e-07	1.40e-02/197/23/4.08e-07	1.13e-02/172/21/7.00e-07
*x*_1_(10)	1.59e-02/121/20/9.24e-07	2.96e-02/245/28/3.26e-08	1.70e-02/156/20/5.03e-07
*x*_2_(10)	1.71e-02/139/23/3.20e-07	2.31e-02/207/24/3.23e-07	1.71e-02/172/21/9.92e-07
*x*_3_(10)	1.64e-02/133/22/4.97e-07	1.71e-02/154/18/5.82e-07	2.13e-02/198/25/4.52e-07
*x*_4_(10)	1.66e-02/139/23/3.20e-07	2.75e-02/253/29/1.30e-07	1.88e-02/172/21/9.91e-07
*x*_5_(10)	1.74e-02/139/23/3.20e-07	2.38e-02/207/24/3.23e-07	1.87e-02/172/21/9.92e-07
*x*_6_(10)	1.07e-02/77/14/4.95e-07	5.20e-03/29/6/6.40e-07	2.00e-02/168/34/8.03e-07
*x*_7_(10)	1.69e-02/139/23/3.16e-07	2.56e-02/224/26/2.58e-07	1.84e-02/172/21/9.76e-07
*x*_1_(50)	5.83e-02/133/22/3.20e-07	7.86e-02/225/26/9.85e-07	5.50e-02/157/21/5.36e-07
*x*_2_(50)	6.06e-02/139/23/7.15e-07	8.53e-02/233/27/9.62e-07	6.04e-02/188/23/4.61e-07
*x*_3_(50)	5.76e-02/133/22/7.93e-07	5.33e-02/144/17/9.57e-07	7.50e-02/229/29/7.42e-07
*x*_4_(50)	5.94e-02/139/23/7.15e-07	7.64e-02/216/25/6.62e-07	6.09e-02/188/23/4.61e-07
*x*_5_(50)	6.09e-02/139/23/7.15e-07	8.66e-02/242/28/9.65e-07	6.29e-02/188/23/4.61e-07
*x*_6_(50)	3.57e-02/83/15/2.35e-07	1.62e-02/35/7/1.44e-08	6.62e-02/178/36/6.13e-07
*x*_7_(50)	5.87e-02/139/23/7.14e-07	9.05e-02/251/29/8.44e-07	6.01e-02/188/23/4.56e-07
*x*_1_(100)	9.06e-02/127/21/8.80e-07	1.36e-01/224/26/5.66e-07	1.01e-01/181/23/1.86e-07
*x*_2_(100)	1.01e-01/145/24/7.00e-07	1.32e-01/217/25/8.82e-07	1.06e-01/188/23/6.52e-07
*x*_3_(100)	9.43e-02/139/23/3.60e-07	1.25e-01/206/24/6.35e-07	1.09e-01/198/25/2.40e-07
*x*_4_(100)	9.89e-02/145/24/7.00e-07	8.15e-02/126/15/6.17e-07	1.01e-01/188/23/6.52e-07
*x*_5_(100)	1.00e-01/145/24/7.00e-07	1.36e-01/217/25/8.82e-07	1.03e-01/188/23/6.52e-07
*x*_6_(100)	6.24e-02/83/15/3.32e-07	2.97e-02/35/7/2.04e-08	1.11e-01/178/36/8.68e-07
*x*_7_(100)	1.02e-01/145/24/6.99e-07	1.44e-01/242/28/1.22e-07	1.02e-01/188/23/6.47e-07

**Table 8:** Numerical results for Problem 8.

	HCGP	HSDY	DCG
Inti(*n*)	CPUT/NFE/NI/NORM	CPUT/NFE/NI/NORM	CPUT/NFE/NI/NORM
*x*_1_(1)	1.39e-04/4/1/0.00e+00	8.44e-04/34/11/8.11e-07	7.05e-05/4/1/0.00e+00
*x*_2_(1)	4.67e-04/22/7/1.01e-08	1.31e-03/51/17/0.00e+00	2.65e-03/124/21/4.07e-07
*x*_3_(1)	4.08e-04/19/6/0.00e+00	2.18e-04/10/3/0.00e+00	1.09e-03/76/13/3.84e-07
*x*_4_(1)	4.59e-04/22/7/1.03e-08	1.33e-03/48/16/1.42e-07	1.59e-03/124/21/4.22e-07
*x*_5_(1)	5.85e-04/22/7/1.01e-08	1.40e-03/51/17/0.00e+00	1.66e-03/124/21/4.07e-07
*x*_6_(1)	1.21e-04/5/1/0.00e+00	1.68e-04/5/1/0.00e+00	9.61e-05/5/1/0.00e+00
*x*_7_(1)	4.82e-04/22/7/1.86e-08	7.30e-04/28/11/0.00e+00	1.62e-03/124/21/8.65e-07
*x*_1_(5)	5.13e-04/4/1/0.00e+00	1.86e-03/12/5/0.00e+00	2.98e-04/4/1/0.00e+00
*x*_2_(5)	3.38e-03/22/7/2.29e-08	7.49e-03/58/19/8.87e-07	1.08e-02/124/21/9.24e-07
*x*_3_(5)	2.29e-03/19/6/0.00e+00	1.25e-03/10/3/0.00e+00	5.58e-03/76/13/3.85e-07
*x*_4_(5)	2.40e-03/22/7/2.30e-08	6.10e-03/51/17/6.16e-07	9.10e-03/124/21/9.31e-07
*x*_5_(5)	2.50e-03/22/7/2.29e-08	7.74e-03/58/19/8.87e-07	8.70e-03/124/21/9.24e-07
*x*_6_(5)	4.80e-04/5/1/0.00e+00	7.08e-04/5/1/0.00e+00	5.66e-04/5/1/0.00e+00
*x*_7_(5)	2.53e-03/22/7/2.55e-08	4.08e-03/34/11/0.00e+00	1.04e-02/136/23/4.20e-07
*x*_1_(10)	8.92e-04/4/1/0.00e+00	2.47e-03/12/5/0.00e+00	4.10e-04/4/1/0.00e+00
*x*_2_(10)	4.67e-03/22/7/3.24e-08	9.75e-03/51/17/8.46e-07	1.55e-02/136/23/4.21e-07
*x*_3_(10)	3.14e-03/19/6/0.00e+00	1.71e-03/10/3/0.00e+00	9.37e-03/76/13/3.86e-07
*x*_4_(10)	4.23e-03/22/7/3.25e-08	8.41e-03/51/17/8.91e-07	1.65e-02/136/23/4.22e-07
*x*_5_(10)	4.90e-03/22/7/3.24e-08	8.52e-03/51/17/8.46e-07	1.58e-02/136/23/4.21e-07
*x*_6_(10)	6.67e-04/5/1/0.00e+00	6.99e-04/5/1/0.00e+00	5.18e-04/5/1/0.00e+00
*x*_7_(10)	4.21e-03/22/7/3.48e-08	8.26e-03/37/14/7.25e-07	1.56e-02/136/23/6.05e-07
*x*_1_(50)	2.83e-03/4/1/0.00e+00	8.42e-03/12/5/0.00e+00	1.19e-03/4/1/0.00e+00
*x*_2_(50)	1.31e-02/22/7/7.26e-08	3.50e-02/53/18/5.05e-09	4.74e-02/136/23/9.42e-07
*x*_3_(50)	1.11e-02/19/6/0.00e+00	5.64e-03/10/3/0.00e+00	2.51e-02/76/13/3.86e-07
*x*_4_(50)	1.19e-02/22/7/7.26e-08	3.45e-02/53/18/5.21e-09	4.86e-02/136/23/9.43e-07
*x*_5_(50)	1.30e-02/22/7/7.26e-08	3.58e-02/53/18/5.05e-09	4.77e-02/136/23/9.42e-07
*x*_6_(50)	2.11e-03/5/1/0.00e+00	2.71e-03/5/1/0.00e+00	1.92e-03/5/1/0.00e+00
*x*_7_(50)	1.35e-02/22/7/7.57e-08	4.09e-02/60/19/2.00e-07	5.26e-02/142/24/9.66e-07
*x*_1_(100)	2.85e-03/4/1/0.00e+00	1.30e-02/12/5/0.00e+00	2.80e-03/4/1/0.00e+00
*x*_2_(100)	1.94e-02/22/7/1.03e-07	5.82e-02/53/18/7.21e-09	8.14e-02/148/25/4.28e-07
*x*_3_(100)	1.74e-02/19/6/0.00e+00	1.13e-02/10/3/0.00e+00	4.41e-02/76/13/3.86e-07
*x*_4_(100)	1.92e-02/22/7/1.03e-07	5.79e-02/53/18/7.33e-09	8.27e-02/148/25/4.28e-07
*x*_5_(100)	1.86e-02/22/7/1.03e-07	5.81e-02/53/18/7.21e-09	8.16e-02/148/25/4.28e-07
*x*_6_(100)	3.78e-03/5/1/0.00e+00	4.39e-03/5/1/0.00e+00	4.31e-03/5/1/0.00e+00
*x*_7_(100)	2.28e-02/22/7/1.09e-07	4.99e-02/36/14/8.63e-08	8.42e-02/148/25/4.56e-07

## Application on sparse signal restoration

In statistical inference and signal processing, solving linear systems of equations that yield sparse solutions poses a significant challenge. These systems are frequently ill-conditioned, which implies that they are highly sensitive to small perturbations in the input data. This characteristic can lead to instability in the solution, which makes it challenging to compute the solution accurately. To address this, a common approach is to formulate an objective function that combines a sparse $\ell_{1}$-norm regularization term and a quadratic $\ell_{2}$-norm error term. This approach ensures that the solution remains sparse while minimizing the reconstruction error. The corresponding optimization problem can be expressed as follows:

$$\min_{v}f(v)=\frac{1}{2}\|w-Av{\|}_{2}^{2}+\eta \|v{\|}_{1},$$ (20)

where $w\in {\mathbb{R}}^{k}$ represents the observed signal, $v\in {\mathbb{R}}^{n}$ is the unknown signal to be estimated, and η>0 is a regularization parameter that controls the balance between the error and the sparsity of the solution. The matrix $A\in {\mathbb{R}}^{k\times n}(k\ll n)$ defines a linear transformation.

To simplify the optimization, we introduce the decomposition:


v=a−b,a≥0,b≥0.

Here, $a_{i}=(v_{i}{)}_{+}$ and $b_{i}=(-v_{i}{)}_{+}$ for all i=1,2,…,n, where $\left(\cdot {)}_{+}=\max\{0,\cdot \right\}$. By using this decomposition, the $\ell_{1}$-norm of *v* can be rewritten as:


$$\|v{\|}_{1}=e_{n}^{T}a+e_{n}^{T}b,$$

where $e_{n}\;=\;{\left(1,1,\;...\;,\;1\right)}^{T}\;\in \;{\mathbb{R}}^{n}$ is a vector of ones. This leads to a reformulation of the original problem (20) as:

$$\min_{a,b}\frac{1}{2}\|w-A(a-b){\|}_{2}^{2}+\eta e_{n}^{T}a+\eta e_{n}^{T}b,\;s.t.\;a\geq 0,\;b\geq 0,$$ (21)

which is a bound-constrained quadratic program. The next step is to express this problem in a form that is more conducive to numerical solution. By defining $x={\left(a,\;b\right)}^{T}$, we can rewrite the problem (21) as:

$$\min_{x}\frac{1}{2}x^{T}Hx+c^{T}x,\;s.t.\;x\geq 0,$$ (22)

where


$$H=(\begin{array}{cc} A^{T}A & -A^{T}A\\ -A^{T}A & A^{T}A \end{array}),\;c=\eta e_{2n}+(\begin{array}{c} -A^{T}w\\ A^{T}w \end{array}).$$

Since *H* is positive semi-definite, the problem (22) represents a convex quadratic program, which guarantees the existence of a unique optimal solution. Xiao et al. [34] showed that this quadratic program can be transformed into the following simpler form:

e(x)=min{x,Hx+c}=0. (23)

They further demonstrated that e(·) is a continuous and monotone function, which provides useful properties for solving the optimization problem. Consequently, solving the original problem (20) is equivalent to solving the simpler problem (23), which can be expressed in the form of the problem (1). Thus, the proposed method efficiently solves the sparse signal restoration problem.

In this experiment, we compare the performance of the HCGP method against the HSDY and DCG methods. The goal is to restore a sparse signal of length *n* from an observed signal of length *m*. The mean squared error (MSE), used to evaluate the quality of the restoration, is defined as:


$$\text{MSE}:=\frac{1}{n}\|\hat{v}-v{\|}^{2},$$

where $\hat{v}$ and *v* represent the original signal and the recovered signal, respectively. The dimensions of the signal is set to *n* = 6144, *m* = 1536, and *k* = 192 non-zero elements are randomly selected. The matrix *A* is randomly generated in MATLAB, and $w=Av_{0}\;+\;\xi$ is the observed data where ξ is the Gaussian noise. The value *η* is obtained by the same continuation technique for the tested methods, i.e., $\eta =0.1\;*\;\|A^{T}w{\|}_{\infty }$. The initial point is selected as $v_{0}=A^{T}w$ and the numerical experiments terminate when the relative change between successive iterative falls below 10^−5^. The stopping criterion for all methods is defined as:


$$\frac{\left|f_{k}-f_{k-1}\right|}{\left|f_{k}\right|}\leq 10^{-6},$$

where *f*_*k*_ denotes the function value *f*(*v*) at $v_{k}$.

**Fig 4:**
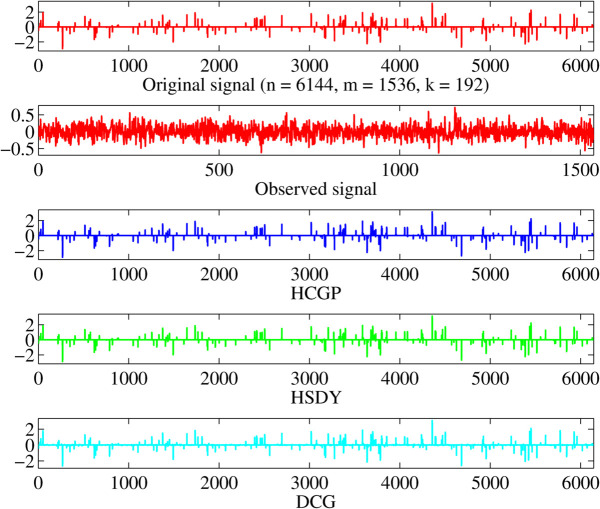
From top to bottom: the original signal, the observed signal, and the recovered signals by HCGP, HSDY, and DCG methods.

**Fig 5:**
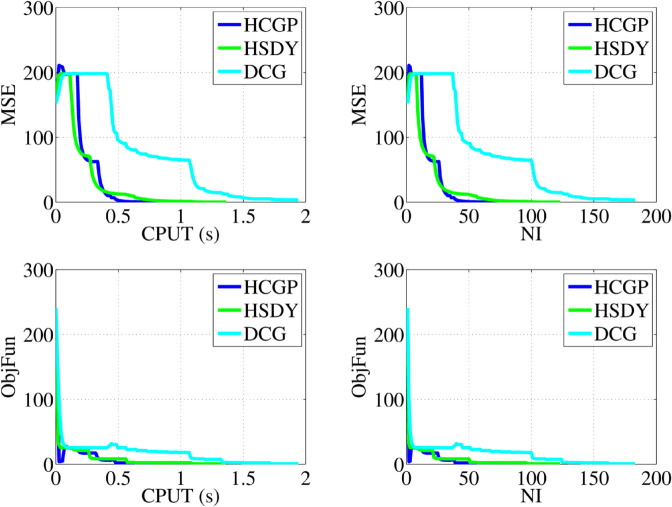
The comparison results of HCGP, HSDY, and DCG methods. The *x*-axis represents the number of iterations (top right and bottom right) and the CPU time in seconds (top left and bottom left). The *y*-axis represents the MSE (top left and top right) and the objection value (bottom left and bottom right

The sparse signal restored by all methods is shown in Fig 4, where it is clear that the HCGP, HSDY, and DCG methods are all capable of successfully restoring the original signal. In addition, Fig 5 illustrates the comparative performance of these methods in terms of convergence properties, including MSE, the CPU time (CPUT), the number of iterations (NI), and the values of the objective functions (ObjFun). To further assess their effectiveness, the experiment (as shown in Table 9) is repeated multiple times, allowing for a robust comparison of these methods. Specifically, in terms of NI and CPUT, the HCGP method outperforms the other two methods, requiring fewer iterations and less computational time to recover the signal.

**Table 9:** Ten experimental results of sparse signal restoration for HCGP, HSDY, and DCG methods.

	HCGP	HSDY	DCG
No.	NI/CPUT/MSE	NI/CPUT/MSE	NI/CPUT/MSE
1	69/0.76/7.818e-06	123/1.35/7.762e-06	183/2.00/5.626e-04
2	67/0.72/1.340e-05	116/1.22/1.330e-05	168/1.82/4.727e-04
3	70/0.75/7.094e-06	154/1.67/1.059e-05	147/1.56/1.051e-03
4	76/0.81/8.313e-06	127/1.34/9.001e-06	124/1.30/1.583e-03
5	72/0.82/7.991e-06	166/1.74/1.011e-05	83/0.88/4.032e-03
6	69/0.75/7.134e-06	130/1.37/7.470e-06	178/2.02/5.336e-04
7	65/0.74/8.058e-06	128/1.39/7.943e-06	237/2.48/3.415e-05
8	67/0.74/1.119e-05	113/1.19/1.245e-05	149/1.57/2.000e-04
9	74/0.83/1.174e-05	132/1.38/1.173e-05	87/0.92/4.262e-03
10	144/1.58/1.849e-05	153/1.65/1.053e-05	168/1.82/2.552e-04
Average	77.30/0.85/1.012e-05	134.20/1.43/1.009e-05	152.40/1.64/1.298e-03
SD	23.67/0.26/3.652e-06	17.56/0.19/2.030e-06	46.16/0.50/1.567e-03

## Conclusions

In this paper, we propose an extension of a hybrid conjugate gradient projection method for finding solutions of constrained nonlinear equations, leveraging hyperplane projection and hybrid techniques. The proposed method is distinguished by its low storage requirement and its exclusive reliance on function values, making it both efficient and practical for large-scale problems. The search direction designed in this method ensures the sufficient descent property, effectively eliminating the need for line search methods. Under suitable assumptions, we prove the global convergence of the proposed method. The experimental results, including those for large-scale constrained nonlinear equations and sparse signal restoration, demonstrate that the proposed method is numerically competitive and efficient, which outperforms similar methods from the literature in terms of both convergence rate and computational efficiency.
